# Directional speakers as a tool for animal vocal communication studies

**DOI:** 10.1098/rsos.230489

**Published:** 2023-05-24

**Authors:** Lily Johnson-Ulrich, Vlad Demartsev, Laurie Johnson, Emma Brown, Ariana Strandburg-Peshkin, Marta B. Manser

**Affiliations:** ^1^ Department of Evolutionary Biology and Environmental Studies, University of Zurich, Zurich 8057, Switzerland; ^2^ Interdisciplinary Center for the Evolution of Language, University of Zurich, Zurich 8057, Switzerland; ^3^ Kalahari Research Centre, Van Zylsrus, 8467 Northern Cape, South Africa; ^4^ Department of Biology, University of Konstanz, Konstanz 78464, Germany; ^5^ Centre for the Advanced Study of Collective Behaviour, University of Konstanz, Konstanz 78464, Germany; ^6^ Department for the Ecology of Animal Societies, Max Planck Institute of Animal Behavior, Konstanz 78467, Germany

**Keywords:** parametric speaker, playback experiment, *Suricata suricatta*, acoustic communication, bioacoustics, soundlazer

## Abstract

Audio playbacks are a common experimental tool in vocal communication research. However, low directionality of sound makes it hard to control the audience exposed to the stimuli. Parametric speakers offer a solution for transmitting directional audible signals by using ultrasonic carrier waves. The targeted transmission of vocal signals offers exciting opportunities for testing the diffusion of information in animal groups and mechanisms for resolving informational ambiguities. We have field tested the quality and directionality of a commercial parametric speaker, Soundlazer SL-01. Additionally, we assessed its usability for performing playback experiments by comparing behavioural responses of free-ranging meerkats (*Suricata suricatta*) with calls transmitted from conventional and parametric speakers. Our results show that the tested parametric speaker is highly directional. However, the acoustic structure of meerkat calls was strongly affected and low frequencies were not reliably reproduced by the parametric speaker. The playback trials elicited weakened behavioural responses probably due to the partial distortion of the signal but also indicating the potential importance of social facilitation for initiating mobbing events in meerkats. We conclude that parametric speakers can be useful tools for directed transmission of animals calls but after a careful assessment of signal fidelity.

## 1. Introduction

Playback experiments are a common tool for investigating the ecology and evolution of animal vocal communication [1]. Playbacks allow researchers to verify observed acoustic patterns and test predictions regarding the function of calls and individual communication strategies. Hence, they have significantly contributed to the understanding of complexity of animal communication systems, the types of information conveyed in vocal signals, and the acoustic channels bearing such information [2]. The basic technique for performing a playback experiment consists of exposing one or more animals to an audio stimulus and monitoring the subsequent response. The stimulus can be natural (as recorded), manipulated (acoustically, compositionally) or artificially generated [1,3,4]. In most cases, audio signals are presented as non-interactive stimuli, i.e. they are fixed and do not vary according to the behaviour of the subject. By contrast, interactive playback designs include a flexible stimulus that is dynamically altered according to the subjects' responses [1,5]. Regardless of the experimental design, which is dependent on the specificities of the study system, the general motivation for conducting playbacks is to verify an observed phenomenon and test the causal relationship between the acoustic stimuli and the response of the receiver. The availability of portable and high-quality audio equipment for both recording and playing sound has made audio playbacks one of the most widespread types of experimental manipulation in field conditions [6].

Vocalizations are an important source of social information for animals [7–9], and exchange of information is hypothesized to be one of the key selective forces for the evolution of social living [10–13]. Conventional speakers usually produce roughly radial sound propagation, and their range is determined by multiple parameters including intensity at the source, frequency composition, medium of transmission, levels of background noise and the audible sensitivity of the potential receivers [14,15]. When these variables are combined with non-homogeneous environmental conditions and structure, predicting the spatial reach of a transmitted signal and fully accounting for receiver exposure to the transmitted playback can be difficult. The inability to control for or accurately determine the number and/or the identities of potential recipients is a limitation to experimental designs that investigate the pathways by which information spreads within animal groups.

With the possibility of transmitting a narrow and precisely directed acoustic beam, experimenters would be able to change the information state of selected individuals within a group. Subsequently, researchers could then track how the target individuals’ response affects the behaviour of non-target individuals, what part of the group ends up being affected, how quickly the information spreads and how long the effect lasts [16,17]. These questions could be tested both qualitatively (comparing between different signal types and traits of targeted individuals) and quantitatively (varying the signal intensity and the number of targeted individuals). Another intriguing possibility is the simultaneous introduction of differential information to different individuals or different sections of an animal group. This design would allow researchers to test how various signals are weighed against one another and assess individuals' reliability and influence over group decisions. These kinds of experiments are not uncommon in the investigation of visual information transfer through natural or experimentally induced behaviours [10,18–23], but technological limitations have prevented such designs in animal vocal communication studies. Currently, directing a stimulus towards specific individuals is only possible in spatially dispersed groups and using proportionally low-intensity signals, while it is nearly impossible to perform such experiments in tightly cohesive groups and with high-intensity calls.

The ability to control and limit the propagation of an acoustic signal would also allow researchers to reduce the number of unaccounted bystander playback exposures. Generally, playback procedures are considered minimally intrusive and, in most cases, do not cause negative behavioural effects or other animal welfare-related issues [24,25]. Nevertheless, several reports suggest that even a single exposure to a playback stimulus is sufficient to generate long-term memories and modify behaviour during subsequent exposures [26,27]. Conversely, multiple and frequent exposures to playback stimuli can cause habituation and reduce responsiveness to similar signals, both artificial and natural. This habituation can affect the validity of experimental results [28,29] and also potentially have detrimental effects on individual fitness and survival [30]. To minimize such effects and comply with ethical requirements on animal experimentation, playback procedures are often limited in their number of repetitions and potential recipients. The use of targeted playbacks would allow researchers to closely control all potential recipients even in studies where information transmission is not the primary focus.

An intriguing solution to the above-mentioned limitations is the use of parametric speakers for targeted playbacks. Parametric speakers have existed for almost four decades [31]; however, they have only become widely commercially available in the last decade. These speakers can produce sound directionality of ±30° from the centre axis [32] and sound pressure values are reduced by 9 dB at only ±15° from the centre axis [33]. In comparison, most conventional speakers have almost uniform 360° sound radiation (at 1 kHz) [34]. The technical specificities by which parametric speakers achieve sound directionality are beyond the scope of this paper and were thoroughly described in the literature over the last decades [31,35–37]. Briefly, an array of piezoelectric transducers is used to deliver a series of ultrasonic waves forming a narrow beam. When this beam encounters a physical obstacle, a secondary spectral component is generated at the difference frequency of the ultrasonic waves, resulting in a highly directional, sonic (audible) signal [38]. In recent years, parametric speakers have received multiple applications and their physiological safety to humans have been demonstrated [32,39–41]. However, despite the potential applicability and impact on experimental practices in animal vocal communication research, no reports on testing such systems are found in the existing literature.

In this work, we field tested a compact parametric speaker, the Soundlazer, SL-01 (Telespial Systems, Inc, CA, USA) in comparison with a conventional (low directivity) speaker, Braven BRV-1 (ZAGG Inc, UT, USA). We performed a series of controlled playback experiments using free-ranging meerkats (*Suricata suricatta*) as a model system. Meerkats are social mongooses native to the Kalahari Desert in Southern Africa [42,43]. Their social and communication systems have been extensively studied in the last decades (reviewed in [44,45]). Meerkats live in cooperatively breeding groups ranging from 2 to 50 members [46,47] and have a large vocal repertoire of approximately 30 call types that have been defined by their function and context of emission. Vocal communication is highly important in meerkat groups both for group cohesion and predator avoidance. Researchers have identified calls that facilitate group cohesion while foraging and during movement [48,49], predator-specific and more general alarm calls, sentinel warning calls [50] and recruitment calls for direct predator cues [51]. Previous studies investigating meerkat vocalizations have used traditional playback procedures [52–56] and serve as a reference for expected behavioural responses to these various call types. The cohesiveness of meerkat groups, where animals typically forage 0–20 m (average about 5 m) from each other [49], and their reliance on vocal communications makes them a suitable system for testing the directionality of a parametric speaker in the field and its ability to reproduce biologically relevant acoustic signals.

The main aim of this work was to test the practical feasibility of using a parametric speaker for delivering targeted acoustic stimuli in an animal's natural habitat. To assess feasibility, we tested: (i) the directionality of the sound beam and the ability to target selected individuals in a closely spaced social group and (ii) the ability of the system to deliver a signal that is informationally whole and elicits a biologically relevant response fitting to the natural behaviour of the receivers. To satisfy these two requirements, the parametric speaker must elicit a similar behavioural response as the conventional speaker. However, the affected individuals should be only the ones positioned directly in line with the axis of the parametric speaker.

**Figure 1.**
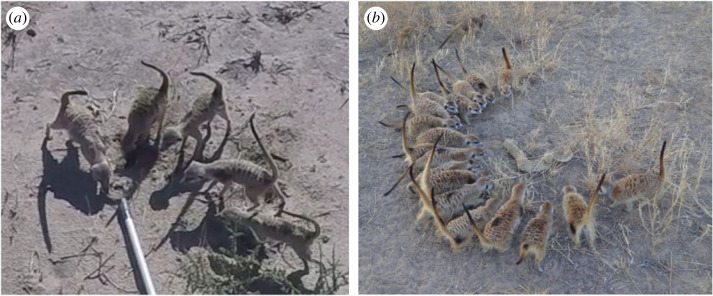
Meerkats mobbing a secondary predator cue (cat urine) (*a*) and a snake (*b*). Reproduced from Driscoll *et al*. [57].

**Figure 2.**
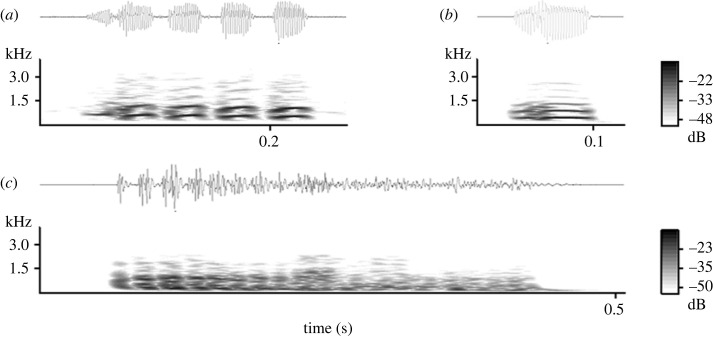
Waveform and spectrograms of three meerkat call types: (*a*) close call, (*b*) low-urgency recruitment call, (*c*) high-urgency recruitment call. Spectrograms were generated in Avisoft-SASLab Pro, FFT length = 1024, Hamming window, resolution = 47 Hz, overlap = 96%.

For our audio stimuli, we used playback tracks of recruitment calls. These calls are produced when a meerkat encounters stationary animals on the ground or in a hole (snakes, jackal, foxes or foreign conspecifics), direct predator cues (hair, faeces, urine) or deposits of foreign conspecifics [50–52]. In response to recruitment calls playbacks, meerkats typically express a mobbing-like response (figure 1) by approaching the loudspeaker with erected fur and tails [50]. Recruitment calls can express two types of urgency (high and low) that probably reflect the affective state of the caller (figure 2*b,c*) [50]. Recruitment calls are therefore an ideal test stimulus because they should elicit a very clear, group-wide behavioural response in meerkats.

As control stimuli, we used short playback tracks of close calls. These calls (figure 2*a*) are the most frequently emitted call type in the meerkat vocal repertoire and are produced during group foraging behaviour. Close calls appear to primarily function as a cohesion mechanism to avoid separation from the group [48,49]. Individual animals' call rate is dependent on their spatial position relative to conspecifics [49]. Call structure includes both individual and group signatures [54,58], and varies based on behavioural context [59]. We chose close calls playbacks as our control stimuli because the production of close calls within a group is very frequent (six calls min^−1^) [60] and close calls only elicit minimal approaches from meerkats (e.g. cohesion effects).

In response to recruitment calls playbacks, we predicted that meerkats would approach the conventional speaker from all directions (on- and off-axis) while the parametric speaker should only stimulate approach from individuals in front (on-axis) of the speaker. Close calls should elicit only mild to no response.

## 2. Methods

### 2.1. Subjects and study site

This work was performed in August 2021 and September 2022–March 2023, at the Kalahari Research Centre in Kuruman River Reserve, Northern Cape, South Africa. The wild meerkat population at the site consisted of fully habituated and individually marked social groups. The animals were accustomed to the presence of human observers and the appearance of novel research equipment.

### 2.2. Audio equipment

Two speakers were used during this study: a conventional speaker (Braven BRV-1, ZAGG Inc, UT, USA), powered by an internal rechargeable battery and a parametric speaker (Soundlazer, SL-01, Telespial Systems, Inc, CA, USA) powered by and external 12V AC power bank (Omni 20+, OmniCharge Inc, CA, USA). The speakers were fixed to custom-built wooden stands matching the height of the sound transducers to 10 cm above ground and connected via a 3.5 mm audio cable to an android tablet (Galaxy Tab A7, Samsung Electronics, Korea).

### 2.3. Playback stimuli

**Figure 3.**
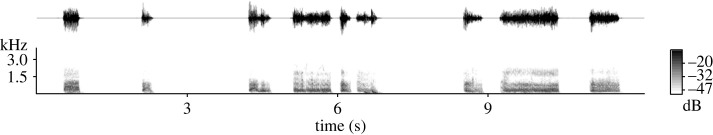
Waveform and spectrogram of a test phase segment from a high-urgency recruitment call playback track. FFT length = 1024, Hamming window, resolution = 47 Hz, overlap = 0%.

A series of audio tracks were designed for this study. Each track included 10 s of silence as an internal technical control, a 10 s playback phase with meerkat calls (figure 3) and an additional 30 s of silence at the end (electronic supplementary material, audio track S1 with recruitment calls). The last silent segment was included in order to clearly and consistently time the experimental window during which behavioural responses were recorded for each of the playback trials. Each track's playback phase included either a series of close calls or recruitment calls with natural background noise in between the calls. Close calls were previously recorded at the tested group (as close calls include individual caller and group signatures [54,58,61]). Recruitment calls were taken from the long-term Kalahari Meerkat Project sound database. Previous research showed no evidence for individual recognition in alarm calls [62], and there is no evidence that caller identity affects the response to recruitment calls (V.D. 2019–2022, L.J.-U. 2021–2022 and M.B.M. 1995–2022, personal observations). The recruitment call tracks included high-urgency calls, low-urgency calls or a combination of both (electronic supplementary material, table S1). All tracks were constructed using Avisoft-SASLab Pro 5.3.00 (Avisoft Bioacoustics e.K, Germany). A high-pass finite impulse response (FIR) filter of 0.3 kHz was applied to the complete tracks to reduce the levels of low-frequency noise, and the track amplitude was normalized.

### 2.4. Technical tests

To assess the technical performance of the parametric speaker, we performed a series of playback and recording tests using both meerkat calls and artificially generated sounds. As our main goal was to establish field usability of the equipment, all tests were done outdoors under conditions similar to those of the experimental set-up. In all tests, we positioned speakers on the ground on a flat sand patch with sparse vegetation, simulating a foraging meerkat. We used a Marantz PMD661Mk2 (Marantz, Japan) digital audio recorder and Sennheiser ME66 (Sennheiser, Germany) directional microphone with a Rycote windshield (Rycote Microphone Windshields Ltd, UK) that was hand held at the height of the speaker at a horizontal distance of 5 m from the speaker. All recording tests were done at 48 kHz sampling rate and 16 bit WAV file format. To minimize low-frequency background noise, a high-pass filter of 0.1 kHz was applied to all recorded files prior to analysis.

#### 2.4.1. Frequency response

To estimate the speakers' frequency responses, we played a 60 s sweep (100–15 000 Hz) twice from each speaker and recorded the on-axis output at a 5 m distance. The frequency contour and the amplitude envelope were extracted in Avisoft-SASLab Pro.

#### 2.4.2. Directionality

To compare the directionality of the playback speakers, we played meerkat recruitment calls from both speakers and recorded the output at a 5 m distance on-axis as well as at ±3 m off-axis. The same audio track was used for all recordings. We manually labelled the calls in Avisoft-SASLab Pro and extracted mean RMS values (root mean square in dBFS) from each call using built in Avisoft functions. We then subtracted the off-axis RMS measurements of each call from the corresponding on-axis measurements to get a measure of relative reduction of loudness between the angular positions.

#### 2.4.3. Sound reproduction quality

To estimate the ability of the playback speakers to maintain the spectral structure of the input signal, we performed a series of recordings using three types of meerkat calls (having different acoustic structures) played from both speakers at a 5 m distance on-axis. We used meerkat high-urgency recruitment calls, low-urgency recruitment calls, and close calls (i.e. the same call types we later used in the animal experimental trials). We manually labelled the call times in the source recordings as well as in the recorded files in Avisoft-SASLab Pro. To compare how well the structure of the input signal was reproduced by each one of the playback speakers, we extracted a series of basic acoustic features from each call: call duration, length of the call in seconds; peak frequency, frequency with the maximum power; *F*_0_, fundamental frequency; *F*_min_, minimal frequency crossing the −20 dB threshold; spectral centroid, the weighted mean of the spectrum; entropy, Wiener entropy as an estimate of sound tonality. These features were selected based on their simplicity and robustness of measurements in order to allow speaker performance comparison and were not intended to comprehensively describe the acoustic structure of the call types used. For all spectral parameters, the mean of all instantaneous spectra between the start and end of the element was internally calculated by Avisoft-SASLab Pro. To statistically compare the selected acoustic features, we performed a correlation test among features extracted from the source track, a recording of conventional speaker and a recording of parametric speaker. We also calculated 95% confidence intervals for the median difference from the source track for each acoustic feature, speaker and call type.

Additionally, we estimated the similarity of the spectrograms by performing a cross-correlation analysis [63] using the ‘xcorr’ function, package ‘WarbleR’. This method compares the spectrogram directly and does not rely on a pre-selected set of acoustic features. The source recording was used as a reference for pairwise correlation with spectrograms of respective calls as recorded from each one of the speakers.

#### 2.4.4. Speaker loudness

Prior to experimental use, we measured the track loudness, from both the parametric speaker and conventional speakers, at the source (less than 0.5 m) and at 5 m distance using a Reed R8050 sound meter (Reed Instruments, NC, USA) using: ‘Fast’ (125 ms) acquisition, C weighting and ‘Lo’ range settings. Under similar settings the output signal of the parametric speaker appeared to be consistently quieter for all playback tracks (also see frequency response figure 5). To streamline experimental procedures, we prepared two versions for each playback: a PS version, used for parametric speaker playbacks, and a CS version in which the source signal was reduced by 15 dB, used for conventional speaker playbacks. This track design allowed us to play both tracks using constant volume settings of the speakers and audio players and maintain output levels that matched to the previously reported mean sound pressure levels (SPL) of naturally emitted meerkat recruitment and close calls [64].

### 2.5. Playback track selection

With our first playback trials, we used only high-urgency recruitment calls (Track 1, electronic supplementary material, table S1). However, given the initial weak responses to the playbacks with the parametric speaker we revised the track design and constructed two new versions that were used in the subsequent playbacks (electronic supplementary material, table S1). Track 2 included only low-urgency recruitment calls with higher mean fundamental frequency (420 Hz) than high-urgency recruitment calls (300 Hz) (figure 2). We hypothesized that higher frequency signals would be more reliably reproduced by the parametric speaker. However, when we tested the low-urgency Track 2 with the conventional speaker, meerkats showed a milder behavioural response in comparison with the high-urgency Track 1 with the conventional speaker (Track 1 response proportion = 1.00; Track 2 response proportion = 0.33). Therefore, we constructed a third track that used a selection of the highest frequency recruitment calls (high- or low-urgency) available (mean *F*_0_ = 340 Hz, Track 3). The behavioural response to Track 3 was improved relative to Track 2 (proportion approaching = 0.65). Therefore, Track 3 (mixed urgency) was used for all subsequent playbacks with the parametric speaker (electronic supplementary material, table S1).

For separating the potential effects of signal distortion by the parametric speaker on meerkat playback responses from the potential effects caused by the signal directionality (fewer receivers), we prepared two additional control tracks (CS-control) *post hoc*. We recorded the output of the parametric speaker transmitting Track 1 and Track 3 and used these secondary recordings in a subset of *post hoc* conventional speaker trials (for a more detailed explanation about *post hoc* hypotheses see Results: Noise distortion versus social facilitation hypotheses).

We used the same recruitment call track regardless of the group the playback was performed in because there is no evidence that recruitment calls contain individual or group signatures. However, close calls do contain group-specific signatures and other contextual information [54,58,59]. Therefore, we only used close calls from meerkats currently living in each group for close call playback tracks.

### 2.6. Playback experiments

We performed playback trials in the morning (8.00–11.30) and in the afternoon (15.00–19.00), when meerkats foraged away from their sleeping burrow. We sampled groups opportunistically while maintaining the criteria of greater than 7 days interval since the preceding experimental manipulation (audio playback or other intervention) to avoid habituation. Each meerkat group was sampled one to three times. Playbacks were only conducted if the group had been foraging continuously for at least 5 min and at least 5 min after any alarm events or other disturbances [52,55].

**Figure 4.**
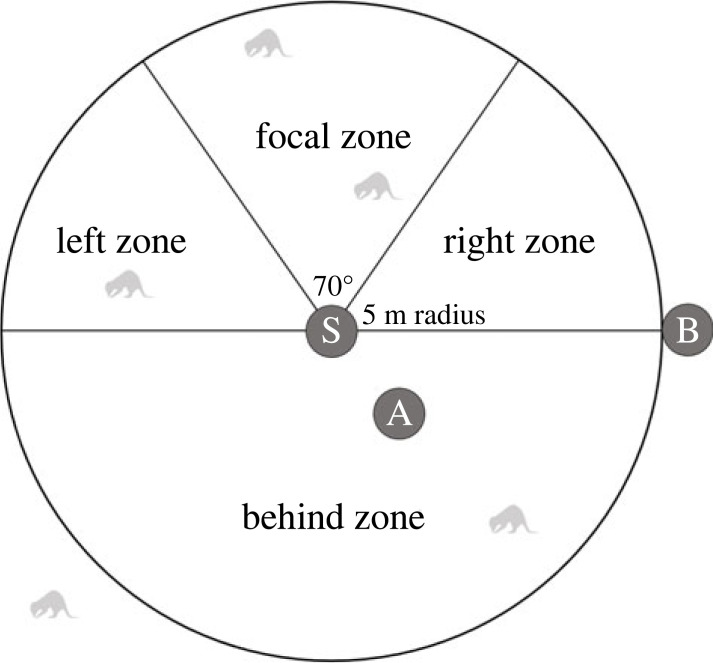
Visualization of testing set-up from above. The relative locations of the observers are indicated by circles (A and B), the speaker location is indicated by a circle (S). Grey meerkats represent possible meerkat positions at the start of a trial. Only the responses of meerkats within the 5 m radius of the sampling zone and any meerkats that subsequently entered the 5 m radius were recorded. Meerkat visualization adapted from a photograph by Bernard Dupont (CC BY-SA 2.0).

We defined the experimental arena as a 5 m radius circle with the speaker positioned in the centre (figure 4). The on-axis focal zone was defined as an approximately 70° triangle in front of the speaker. We chose playback locations by identifying a ‘sampling circle’ in which we could successfully target at least one focal (on-axis) individual and at least one non-focal (off-axis) individual and all meerkats were at least 2 m away from the speaker. Non-focal meerkats were categorized as in a ‘side’ zone if they were between the focal zone and the perpendicular axis to the speaker, and as in the ‘behind’ zone if they were behind the perpendicular axis (figure 4). All playbacks were filmed using one or two wide-angle action video cameras GoPro black 7 and 8 (GoPro Inc, CA, USA). The speaker operator was positioned approximately 1 m behind and off-axis of the speaker and filmed the behaviour of meerkats in the focal (on-axis) zone with camera one. A second observer was positioned outside the sampling circle on the perpendicular axis and filmed the behaviour of meerkats in the non-focal (off-axis) zones with camera two. The behaviour of meerkats outside the sampling circle were not recorded (electronic supplementary material, videos S1–S7).

We performed one to four playback trials per session. In general, we attempted to perform two recruitment call playbacks with the parametric speaker each session and one or two additional control playbacks of a close call control track, with parametric speaker, the conventional speaker or both. Overall, because responses to recruitment call playbacks are already well documented [50–52], we aimed to conduct only one recruitment call playback with the conventional speaker per meerkat group. Within sessions, there was some variability in the number and type of playbacks based on external circumstances (e.g. strong winds, alarm call interruptions). All playbacks were separated by at least 10 min. Because recruitment call playbacks with the conventional speaker typically generate a very strong response from meerkats, including those outside the sampling zone, these playbacks were only conducted once per session and at the end of a session.

### 2.7. Behavioural scoring

Each meerkat's behaviour was scored from video as a binary response indicating whether they approached the speaker or not. An approach was defined as moving at least 1 m towards the speaker. Approaches were counted if they occurred during the 10 s playback phase or the terminal 30 s silent segment of the track. All videos were scored independently by both L.J.-U. and V.D. for the counts of focal versus non-focal meerkats present and the counts of focal versus non-focal meerkats that approached the speaker (*n* = 41 playbacks × 4 counts per playback = 164 observations). The overall per cent agreement was 81.1% with a Fleiss kappa indicating ‘substantial agreement’ (*κ* = 0.746, *z* = 17.4, *p* = 0) and with a highly significant Spearman's correlation test (*p* = 0.93, *p* < 0.001). Overall, L.J.-U. and V.D. had perfect agreement on counts for 133/164 observations, differed by one meerkat for 27 observations, and differed by two to four meerkats for only four observations. For all counts that differed by more than one meerkat, L.J.-U. and V.D. re-coded the videos together for unanimous agreement prior to statistical analysis.

### 2.8. Statistical analysis

All statistical analyses were conducted in R Studio (R version 4.1.2 and R Studio Build 372) [65,66]. All figures (except for spectrograms/waveforms) were produced using ggplot2 [67]. We used binomial regression (link = logit) to analyse the proportion of meerkats approaching the speaker per playback experiment. As predictor variables we included call type (recruitment versus close call) and condition (conventional speaker, parametric speaker focal or parametric speaker non-focal (model 1)).

While we recorded all meerkats as belonging to either front, side or behind zones for both the conventional and parametric speakers in our videos and raw data, for the analysis we merged these three categories for the conventional speaker because sound propagates equally in all directions with the conventional speaker. For the parametric speaker, we also merged the side and behind zones because there appeared to be no difference in responses between these two categories (electronic supplementary material, figure S1). This resulted in three levels for variable ‘condition’: conventional speaker (CS), parametric speaker focal (PS-focal) and parametric speaker non-focal (PS-non-focal).

For model 1, we next calculated pairwise contrasts using the R package ‘emmeans’ [68]. There were six contrasts that we planned to investigate *a priori* in order to test our predictions. We expected our findings to replicate previous research on recruitment calls and close calls; a significantly larger proportion of meerkats should approach the speaker in response to recruitment calls than close calls for both (i) the conventional speaker and (ii) focal meerkats with the parametric speaker. (iii) A significantly larger proportion of focal meerkats should approach the parametric speaker than non-focal meerkats with recruitment calls. (iv) There should be no significant difference in the proportion of focal meerkats approaching the speaker in response to recruitment calls with the conventional speaker compared with the parametric speaker. For close calls, we generally expected a weak response across all conditions including focal meerkats with the parametric speaker, but (v) there should be close to zero response for non-focal meerkats with the parametric speaker. In addition, (vi) there should also be no significant difference in the proportion of focal meerkats approaching the speaker in response to close calls with the conventional speaker compared with the parametric speaker.

Model 1 showed quasi-complete separation for non-focal meerkats' response to close call playbacks with the parametric speaker (0/21 meerkats approached). As a result, the estimates for this category (close calls, parametric speaker, non-focal meerkats) are unreliable. We report results from model 1 in the main text, but we also created a second model (model 1.2) to confirm that the presence of quasi-complete separation was the cause of unreliable estimates. Here, we manually changed one of the zeros in this category to a one, which changed the raw proportion of meerkats approaching to 1/21 meerkats. Model results for all other categories were largely unchanged, so quasi-complete separation did not appear to influence estimates for all other categories.

We also created two additional models *post hoc* to investigate the effects of social facilitation and possible noise distortion by the parametric speaker on the response to recruitment calls (see Results: Noise distortion versus social facilitation hypotheses). We first created a model looking at condition with four levels (model 2): conventional speaker (CS), parametric speaker focal (PS-focal), parametric speaker non-focal (PS-non-focal), and conventional speaker control (CS-control). The level ‘CS-control’ was an additional playback condition added *post hoc* that used a recording of recruitment call tracks 1 and 3 produced by the parametric speaker and presented to meerkats with the conventional speaker. This additional playback condition was motivated by the results of the initial round of playbacks and is further discussed below. Briefly, the purpose of this additional condition was to separate the potential effects of noise distortion of the parametric speaker (which might lead meerkats not to recognize playbacks as calls) and social facilitation (which might lead meerkats to avoid responding due to a perceived lack of response from other meerkats) in explaining low responses to recruitment calls played from the parametric speaker. Finally, to further explore the role of social facilitation in driving responses to recruitment calls, we created a third model (model 3) with the addition of a group size variable for the total number of meerkats within 5 m (including meerkats that entered the 5 m sampling radius after the start of the playback).

We included random effects for playback trial and group to account for the structure of the data and control for any between-group variation in responses in all models. All models were built using the R package ‘glmmTMB’ [69]. After creating a model, we calculated a dispersion ratio to check for overdispersion using the R package ‘performance’ [70]. Residuals were examined using the R package ‘DHARMa’ [71].

## 3. Results

### 3.1. Technical tests

#### 3.1.1. Frequency response

**Figure 5.**
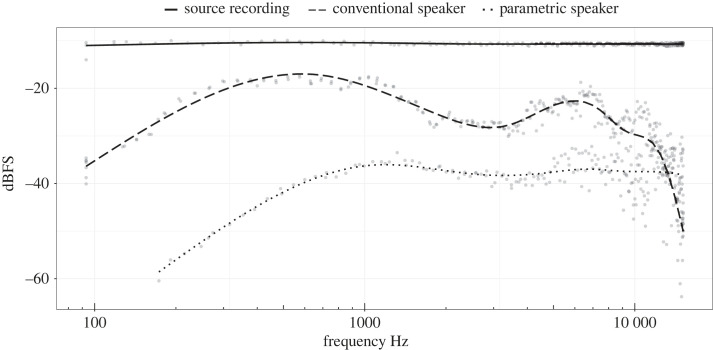
An estimation of playback speakers' frequency response in comparison with the amplitude envelope of the source signal. The source signal is a 60 s, 100–15 000 Hz sweep of constant amplitude. The curves represent generalized additive model (GAM) smoothed frequency and amplitude measurements of the source signal and the speaker outputs, as recorded at a 5 m distance. The conventional speaker (dashed line) showed a flatter pseudo-response curve and was more reliable at reproducing the low-frequency range of the sweep, while the parametric speaker (dotted line) output below 173 Hz could not be detected at the recorded distance.

While not being a technically exhaustive investigation, the estimation of speaker frequency response showed reduced capability of the parametric speaker for reproducing low frequencies in comparison with the conventional speaker (figure 5).

#### 3.1.2. Directionality

**Figure 6.**
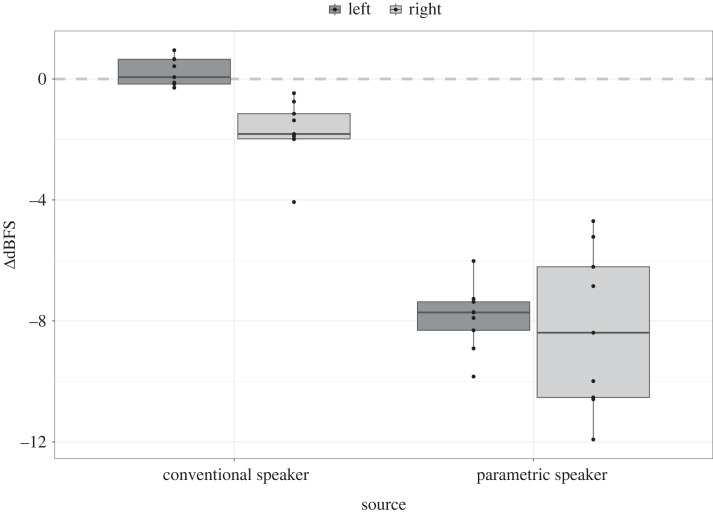
Off-axis relative loudness of the conventional speaker and parametric speaker as recorded by a stationary microphone. Recordings were done at a 5 m distance and at 3 m to the left (dark grey) or right (light grey) from the centre axis of the speaker. *Y*-axis shows ΔdBFS (decibels relative to full scale) values relative to an on-axis (centre) recording which was set as 0 (grey horizontal line).

A comparison of sound directionality between the conventional speaker and the parametric speaker (figure 6) demonstrated a strong decrease in signal loudness at 3 m off-axis with the parametric speaker (mean ± s.d. in comparison with on-axis recording, 9.64 ± 2.16 dB, *n* = 18). The conventional speaker showed a 0.89 ± 0.91 dB decrease between the front and side recording positions (*n* = 18).

The boxplots show the median value of dBFS difference from the on-axis recording (horizontal line), interquartile range (IQR; the box boundaries) and the 95% range (whiskers) of the data. Parametric speaker output showed a decrease in loudness at 3 m off-axis (both to the left and to the right) while the conventional speaker showed little variation between off- and on-axis recordings.

#### 3.1.3. Sound reproduction quality

**Figure 7.**
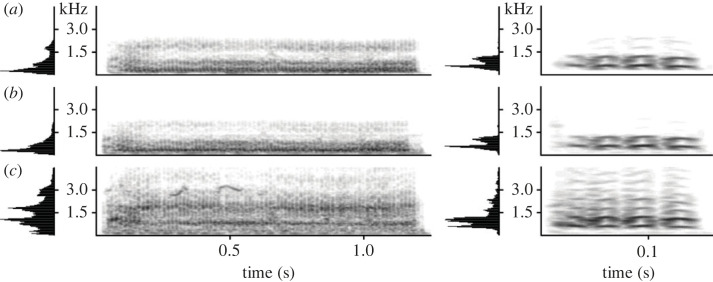
Spectrogram and mean spectrum of meerkat high-urgency recruitment call (left) and close call (right). The panels show the same call exemplars as recorded from different sources: (*a*) recorded directly from a vocalizing animal and used as a source signal for panels *b* and *c*, (*b*) transmitted by the conventional speaker, and (*c*) transmitted by the parametric speaker. The low-frequency recruitment calls are heavily distorted when transmitted by parametric speaker. Recording distance and conditions were identical.

**Figure 8.**
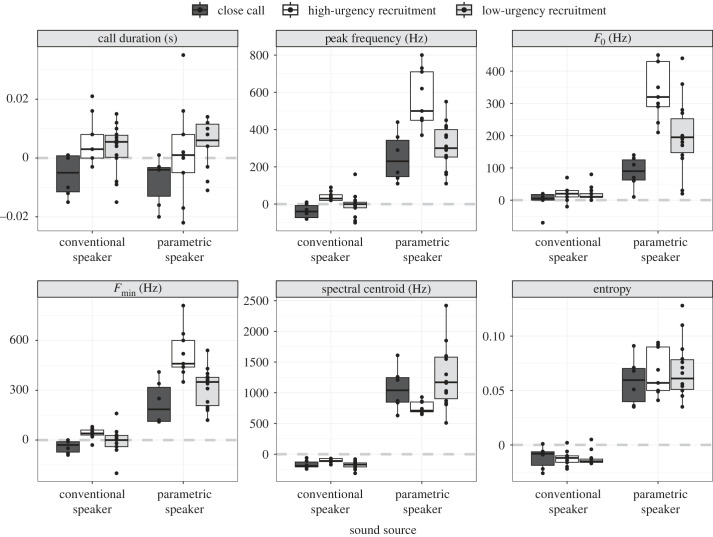
A comparison of reproduction quality of three different meerkat call types (close calls, high-urgency recruitment, low-urgency recruitment) by the conventional speaker and parametric speakers. The measured call duration and spectral parameters were subtracted from the reference values of the respective source recordings (black dots). Grey horizontal line denotes the relative reference level of the respective parameter. *Y*-axis units of measurements are indicated in the subplot headers. The plots show median value (black horizontal line), IQR (the box boundaries) and the 95% range (whiskers) of the data. Per call type sample sizes are: close calls, 6; high-urgency recruitment, 9; low-urgency recruitment, 14). The call duration shows little variation between the two speakers. The spectral measurements of the conventional speaker are in agreement with the source signal, while the parametric speaker demonstrates deviation from the source.

Analysis of field recordings of the playback tracks, played from the two speakers, showed both a qualitative (figure 7) and a quantitative (table 1 and figure 8) discrepancy between the emitted audio signals (electronic supplementary material, file S2). While the conventional speaker was able to reliably reproduce the spectral structure of the original call, audio from the parametric speaker had an upwards spectral shift of the sound, higher entropy of the recorded signal and also showed higher variation in reproduction. With the parametric speaker, all extracted acoustic features, except for call duration, failed to correlate with the source signal (table 1) and showed substantial deviations from the source signal (figure 8). With the conventional speaker, nearly all extracted acoustic features were significantly correlated with the source signal (table 1 and figure 7).

**Table 1.** Pearson correlation between acoustic features of a source audio signal and of a signal transmitted form a conventional and parametric speaker and recording at 5 m distance. Significant correlations of above 0.8 are marked in italics.

		conventional speaker		parametric speaker		
		correlation coef.	*p*-value	correlation coef.	*p*-value	
source recording	recruitment high	*0.9996*	*0.0000*	*0.9985*	*0.0000*	duration
	recruitment low	*0.9059*	*0.0000*	*0.9274*	*0.0000*	
	close call	*0.9880*	*0.0002*	*0.9491*	*0.0038*	
	recruitment high	*0.9457*	*0.0001*	−0.6823	0.0429	peak frequency
	recruitment low	*0.9126*	*0.0000*	0.5608	0.0370	
	close call	*0.8416*	*0.0357*	*0.8645*	*0.0263*	
	recruitment high	*0.9273*	*0.0003*	0.3686	0.3290	*F*_0_
	recruitment low	*0.8275*	*0.0003*	0.2195	0.4509	
	close call	0.7502	0.0858	0.5554	0.2526	
	recruitment high	0.6356	0.0658	−0.5321	0.1404	minimum frequency
	recruitment low	*0.8436*	*0.0001*	0.5683	0.0340	
	close call	0.6600	0.1538	0.7239	0.1038	
	recruitment high	*0.8283*	*0.0058*	0.7522	0.0194	spectral centroid
	recruitment low	0.6492	0.0120	0.6155	0.0191	
	close call	*0.8175*	*0.0469*	−0.5212	0.2890	
	recruitment high	*0.8271*	*0.0059*	0.1711	0.6597	entropy
	recruitment low	*0.9390*	*0.0000*	−0.0880	0.7648	
	close call	*0.8129*	*0.0492*	−0.0619	0.9073	

The spectrogram cross-correlation analysis showed high pairwise correlation between spectrograms of the source recording and of the conventional speaker (mean ± s.d. = 0.94 ± 0.04) in comparison with correlation between spectrograms of the source recording and parametric speaker (mean ± s.d. = 0.78 ± 0.04).

### 3.2. Behavioural experiments

We performed playbacks with 12 different meerkat groups over 21 independent sessions for a total of 41 playbacks with 158 meerkats. On average, we conducted 1.95 ± 1.32 (mean ± s.d.) playbacks per session. Within the sampling circle we recorded 3.85 ± 1.85 meerkats on average, of which 1.61 ± 1.02 were focal meerkats.

**Figure 9.**
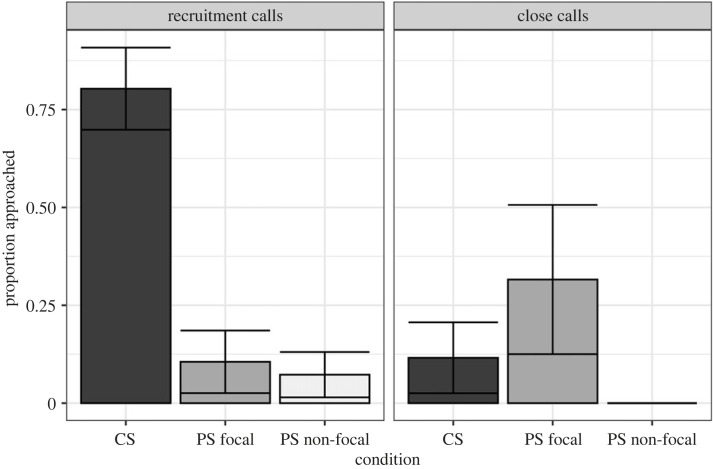
Estimated marginal means for meerkat responses (proportion) to close calls and recruitment calls. Error bars show standard error. CS: conventional speaker; PS focal: parametric speaker, on-axis; PS non-focal: parametric speaker, off-axis.

Model 1 explained a significant amount of variation in responses to playbacks (*χ*^2^ = 30.12, *p* *<* 0.001; electronic supplementary material, table S2). Our results replicated earlier work with conventional speakers (figure 9); the probability of a meerkat approaching the speaker during recruitment call playbacks was 80.34% compared with only 11.59% during close call playbacks (*β* = 3.44, s.e. = 1.15, *p* < 0.01; electronic supplementary material, table S3).

The response of meerkats to recruitment call playbacks with the parametric speaker (focal) was significantly reduced compared with those with the conventional speaker (*β* = 3.55, s.e. = 1.13, *p* *=* 0.003; electronic supplementary material, table S3). As a result, we also failed to detect a significant difference between focal and non-focal meerkats (*β* = 0.41, s.e. = 0.96, *p* *=* 0.67; electronic supplementary material, table S3). Likewise, we failed to detect a difference between recruitment call playbacks and close call playbacks with the parametric speaker for focal meerkats (*β* = −1.36, s.e. = 1.21, *p* *=* 0.27; electronic supplementary material, table S3).

For close calls, our results generally supported our predictions (figure 9). We observed no significant difference between the proportion of meerkats approaching the parametric speaker (focal) and the conventional speaker (*β* = −1.23, s.e. = 1.24, *p* = 0.31; electronic supplementary material, table S3). In addition, focal meerkats had a 31.58% probability of approaching the speaker compared with a 0% probability for non-focal meerkats with the parametric speaker (figure 9).

### 3.3. Noise distortion versus social facilitation hypotheses

We developed two *post hoc* hypotheses regarding the weakened response of meerkats to recruitment calls with the parametric speaker: (i) the parametric speaker may distort the acoustic features of calls enough to affect the information contained in the signal and alter behavioural response or (ii) a response to recruitment calls may be dependent on social facilitation. In other words, by targeting only a few receivers with the parametric speaker we reduce the chances of stimulating a behavioural response, since focal individuals may not receive enough social feedback from conspecifics to initiate an approach. Social facilitation may be particularly important in collective action contexts such as mobbing, where taking part is likely to be individually risky and the risk may increase if fewer group members participate [72–75].

To disentangle these two hypotheses, we performed an additional set of seven playback experiments with the conventional speaker using a recording of recruitment calls produced by the parametric speaker (CS-control, electronic supplementary material, table S1). The goal of this condition was to retain the (distorted) sound quality of the parametric speaker while removing its directionality. We next created two additional models; model 2 compared these new playbacks with our original conventional and parametric speaker playbacks, and model 3 investigated the effect of group size, including the number of meerkats approaching from outside the 5 m sampling circle, on the probability of responding to recruitment calls.

**Figure 10.**
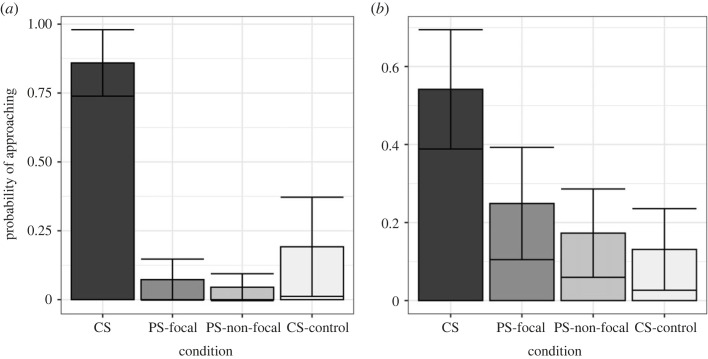
Estimated marginal means for meerkat responses (proportion) to recruitment calls with the conventional speaker (CS), the parametric speaker (PS) and control playbacks of PS recordings with the conventional speaker (CS-control). Error bars show standard error. (*a*) Shows the results from model 2 while (*b*) shows the results from model 3, where group size is included in the model as a control.

**Figure 11.**
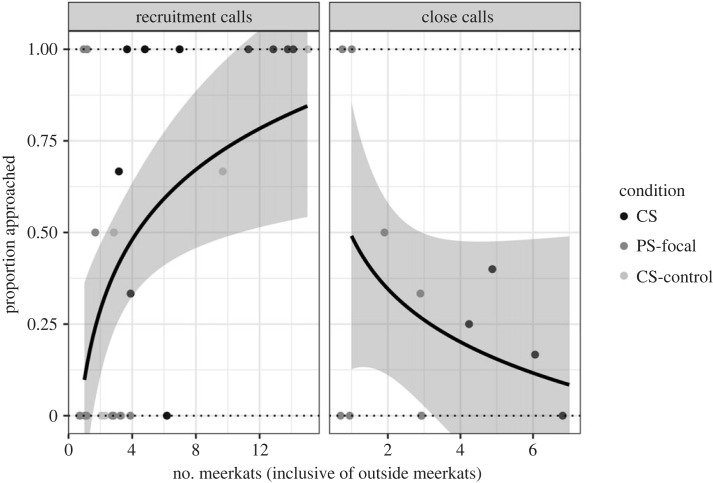
Proportion of meerkats approaching the speaker in response to recruitment calls or close calls (only including meerkats that started within the 5 m sampling radius) as a function of the number of meerkats within the 5 m sampling radius (including meerkats that started within 5 m and those that entered the 5 m radius during the sampling period). CS, conventional speaker; PS-focal, focal meerkats with the parametric speaker; CS-control, conventional speaker playbacks that used recordings from the parametric speaker. Data points show raw proportions, regression lines show logistic curves, and grey bands show 95% confidence intervals fit by ggplot2.

In model 2, we found that response to CS-control playbacks was reduced compared with the conventional speaker (figure 10*a*), but only marginally significantly (*β* = −3.25, s.e. = 1.67, *p* = 0.052; electronic supplementary material, table S6). When we added a group size variable in model 3 (figure 10*b* and figure 11), we found that group size had a significantly positive effect on the probability of responding (*β* = 0.47, s.e. = 0.15, *p* = 0.002; electronic supplementary material, table S7). Furthermore, the difference in the probability of responding to playbacks with the parametric speaker compared with the conventional speaker became non-significant (figure 10*b*). However, for the CS-control playbacks, the effect size and significance remained similar between model 2 and model 3 (*β* = −2.06, s.e. = 1.09, *p* *=* 0.058; electronic supplementary material, table S7).

We also noted that during conventional speaker playbacks a large number of meerkats from outside the 5 m sampling radius often approached the speaker (electronic supplementary material, videos S1 and S2). When we only counted approaches for meerkats that began approaching before any outside meerkats entered the 5 m sampling circle, we observed that only 46% of meerkats within the sampling circle started to approach the speaker prior to any outside meerkats approaching (17/37 meerkats from eight playbacks). However, outside meerkats entered the 5 m radius, on average, 9.5 s after the start of the playback, which is a relatively short time window for the focal meerkat(s) to begin approaching.

## 4. Discussion

Our testing of a parametric speaker showed that it is capable of transmitting a directional and narrow sound beam with approximately 10 dB loudness reduction at 3 m off the central axis. As a comparison, a conventional speaker demonstrated a less than 1 dB loudness reduction under the same testing conditions (figure 5). However, the quality and the spectral structure of the source signals, when transmitted by the parametric speaker, were greatly affected. Frequencies below 500 Hz were less reliably reproduced, resulting in a bandwidth shift towards higher frequencies (figure 7). The tonal structure of the call was also affected by added nonlinearities, resulting in higher entropy of the acoustic signal (figure 8).

Results from playback trials showed that using a parametric speaker for targeted signal delivery is possible, as we were successful in eliciting a significantly stronger behavioural response from on-axis meerkats compared with off-axis meerkats (figure 8). The conventional speaker, on the other hand, had a spatially dispersed effect with similar individual responses documented for both on-axis and off-axis meerkats (figure 8; electronic supplementary material, movies S1 and S2). In regard to delivering a biologically (functionally) relevant signal, we failed to elicit a consistent and predictable recruitment response with the parametric speaker. In contrast, with the conventional speaker, recruitment call playbacks caused multiple individuals to cease their activities and approach the sound source. Meerkat recruitment call functions have been described and tested in the past, demonstrating the robustness of the behavioural responses and providing a strong and convincing argument for its fitness consequences [50–52]. The conventional speaker recruitment call playbacks we performed in this study fully reproduced previous results and provided a behavioural baseline for comparisons.

The results of close call playback trials demonstrated an expectedly mild response with the conventional speaker, in comparison with recruitment calls. Responses to the parametric speaker close call playbacks were also mild and statistically similar to the parametric speaker recruitment call responses. Meerkat close calls serve to maintain group cohesion and are emitted frequently and by multiple individuals [48,49]. Receivers do not demonstrate a very dramatic shift in their behavioural state; nevertheless, they are clearly monitoring their conspecific vocal emissions [54,61]. Meerkats adjust their movement trajectories according to group calling patterns [48] as well as responding to the identities of their immediate neighbours [54]. Thus, the mild and not fully consistent vigilance reaction to the close call playbacks can be explained by context-dependent variation, with some individuals being more motivated to explore the stimulus.

The lack of differential response to the different playback stimuli (recruitment and close calls, figure 8) with the parametric speaker could be explained by two alternative hypotheses that we developed *post hoc*.

First, the signal distortion by the parametric speaker may have disturbed the information bearing channels of the recruitment call. In support of this hypothesis, we found that the parametric speaker poorly reproduced low-frequency sounds which may have rendered the meerkat recruitment calls partly or completely unrecognizable to the animals. We are unable to say with confidence if the calls were perceived as a novel, ‘strange noise’ or whether they were simply ambiguous and below response threshold. Similarly, it is unclear from our results whether the close calls were perceived as biologically relevant signals. We found that 33% of focal meerkats approached the speaker during close call playbacks with the parametric speaker (figure 9). While this proportion was not significantly greater than responses with the conventional speaker, it was much higher than expected for close calls and further supports the results of signal distortion by the parametric speaker (figure 8). Because close calls are individually recognizable by group members, even minor distortion of close calls may have caused them to be perceived as out-group, rather than in-group, calls and prompted a stronger investigatory response.

A second hypothesis is that social facilitation plays a much larger role than previously known in responses to recruitment calls. Social facilitation has been defined as the enhancive or suppressive effect spectators or co-performers have on individuals' behaviour [76]. Previous research on mobbing towards secondary predator cues in meerkats found that the intensity of mobbing correlated with the number of individuals recruited [57], but no research had previously investigated if social facilitation played a role in recruitment itself. When we examined the effect of ‘outside’ meerkats (those that were initially outside the 5 m sampling circle) during conventional speaker playbacks, we found that less than half of all meerkats began their approach towards the speaker before outside individuals approached. This waiting behaviour suggested that some degree of social facilitation does occur and individual meerkats might assess coalitional support before committing to a mobbing event. During parametric speaker playbacks, we never observed outside meerkats approaching, probably as a result of the limited propagation of sound. Therefore, we decided to include a variable for group size that included the number of meerkats originally within 5 m *and* those that entered the 5 m sampling zone during the playback. This group size metric had a highly significant effect on the probability of meerkats (that were originally within 5 m of the speaker) approaching the speaker location following recruitment call playbacks (pooled conventional and parametric speakers, figure 10).

To further distinguish the ‘strange noise’ and ‘social facilitation’ hypotheses, we performed an additional seven playbacks with the conventional speaker using recordings of recruitment calls produced by the parametric speaker (CS-control). If the ‘social facilitation’ hypothesis was true, and it was the small number of receivers rather than the distorted sound of the recruitment calls causing the reduced response to parametric speaker playbacks, we expected to observe a CS-control response similar to that seen with the conventional speaker recruitment call playbacks. By contrast, if the ‘strange noise’ hypothesis was true, we expected to see a reduced response to control playbacks, similar to that observed during parametric speaker recruitment call playbacks.

Based on our follow-up playback experiments, we found an estimated probability of 19.2% to approach the speaker during CS-control trials. This response was higher than the parametric speaker playbacks but not nearly as strong as expected compared with the conventional speaker (figure 9*a*, model 2). After adding group size to this model, the probability of approaching the speaker location for the parametric speaker was no longer significantly different from the conventional speaker (figure 9*b*, model 3), which would suggest that variation in group size explained most of the difference between the conventional and parametric speakers. However, the probability of approaching for the CS-control playbacks remained similar between these two models (figure 9, models 2 and 3). This result shows that, even after controlling for social facilitation, we still observed a relatively low probability of approaching the speaker during CS-control playbacks. Taken together, our results suggest that both the signal distortion and social facilitation could have contributed to the mild recruitment response following the parametric speaker and CS-control playbacks. Further technical and experimental testing is required to understand what acoustic frequencies are most reliably reproduced by parametric speakers and how the reconstruction of audible signal by demodulation of ultrasonic waves affects its fidelity. It is possible that in species with predominantly mid- and high-frequency vocal repertoires the signal distortion would be minimal and allow parametric speaker usage for exploring new and exciting avenues of targeted information delivery and manipulation. Alternatively, a different parametric speaker model or manufacturer may have better capabilities for reproducing low acoustic frequencies. Perhaps speaker models with larger arrays of transducers are more capable of reproducing lower acoustic frequencies than smaller arrays.

Animal vocal signals convey information in various ways, including frequency composition and modulation, signal duration, vocalization rate, syntactic structure and more [77–80]. Often, to increase signal robustness to degradation, information is encoded redundantly in multiple back-up channels [81,82]. Still, signal degradation due to transmission distance, jamming or structural distortion at the source can make it incomprehensible to the receivers [83–85]. Since animal sound perception is different from that of humans, there are limitations on the experimenter's ability to audibly assess the integrity of the transmitted audio signal. Differences in speakers used for playbacks can introduce biases in terms of sound propagation patterns, frequency composition and amplitude mismatch [86]. It is thus pivotal that playback equipment and experimental procedures are documented, calibrated and tested against a clear, natural and study-system-specific response. Our results emphasize the importance of ensuring the flatness of speaker frequency response while paying attention to the bandwidth of the signal to be transmitted. A poor or non-even sound reproduction in the relevant parts of the spectrum could result in the signal becoming unrecognizable by the receivers. Diagnostic equipment and testing facilities are not always available, especially in fieldwork settings. Nevertheless, a series of recording tests and simple comparison between the source and recorded signals can be valuable for flagging potential technical issues in delivering the audio stimulus.

Ultimately, while the parametric speaker we used in this work did not fully reproduce meerkat calls, we demonstrated how the use of highly directional playbacks can uncover previously unknown mechanisms underlying individuals' decision to join group action. Previous research on meerkat recruitment calls was only able to examine the responses of entire groups [52], while with the parametric speaker we were able to target one to three individuals at a time, with the rest of the group remaining unaware of the transmitted stimulus. When we compared the probability of approaching the speaker location with the number of meerkats present within 5 m, we found a strong positive association which suggests that the decision to join a recruitment event is socially facilitated (dependent on the presence and behaviour of neighbouring conspecific). Furthermore, the distribution of proportion of meerkats responding to recruitment calls (electronic supplementary material, figure S3) shows a distinctive U-shaped pattern, which suggests an ‘all or nothing’ collective strategy where either all individuals join the collective action, or no individuals join. Given the potential risks involved in joining the mobbing event, when encountering a live snake or other ground-level threats [50,52], this collective strategy is not surprising. Animals have been shown to adjust their behaviour to the ‘odds’ of the outcome. For example, some female lions (*Panthera leo*) are more likely to join intergroup encounters when the intruders were outnumbered, while others demonstrate opposite tactics and are more likely to join when the intruders were perceived to have an advantage [72]. In spotted hyenas (*Crocuta crocuta*), the initiation of lion mobbing is positively affected by the number of hyenas present and also partly by the number of greeting behaviours [73] which were suggested to reinforce social bonds and facilitate cooperative actions [87]. Thus, the decision to engage in risky group behaviour is dependent on assessment of multiple factors, e.g. the levels of threat, number of group members present and possibly their ‘commitment’ for participating.

Overall, directional playbacks show great promise for future work investigating the mechanisms of collective responses and the thresholds for the formation of joint action events in cohesive animal groups. Future work could investigate in detail the role of leadership versus quorum mechanisms and quantify the contribution of each additional animal to initiation of joint action. Additionally, in systems where individual life-history data is available, the effect of traits, such as age, kinship and rank can be experimentally tested for their relative contribution for initiating collective action.

## 5. Conclusion

In sum, we verified the use of parametric speakers as potential tools for targeted, individual playbacks within a cohesive social species. We also demonstrated how targeting a small subset of individuals can reveal previously ‘hidden’ mechanisms underlying collective responses to potential threats. Overall, we think parametric speakers are highly promising tools for investigating individual and collective decision-making and the diffusion of social information. However, the fidelity of the transmitted signal should be thoroughly tested, especially for species with low-frequency vocalizations (less than 500 Hz). We also anticipate high potential for parametric speakers in studies where precise recognition of the transmitted signal is less important (e.g. testing the effects of novel acoustic stimuli or anthropogenic noise). However, parametric speaker technology is continuously improving and new, more advanced models may be able to address the limitations described here.

## Data Availability

The datasets and R code are available from the Dryad Digital Repository: https://doi.org/10.5061/dryad.gmsbcc2t0 [88].
